# Protamine/heparin optical nanosensors based on solvatochromism†

**DOI:** 10.1039/d1sc04930e

**Published:** 2021-11-15

**Authors:** Yoshiki Soda, Kye J. Robinson, Robin Nussbaum, Eric Bakker

**Affiliations:** Department of Inorganic, Analytical Chemistry, University of Geneva Quai Ernest-Ansermet 30 1211 Geneva Switzerland Eric.Bakker@unige.ch

## Abstract

Optical nanosensors for the detection of polyions, including protamine and heparin, have to date relied upon ion-exchange reactions involving an analyte and an optical transducer. Unfortunately, due to the limited selectivity of the available ionophores for polyions, this mechanism has suffered from severe interference in complex sample matrices. To date no optical polyion nanosensors have demonstrated acceptable performance in serum, plasma or blood. Herein we describe a new type of nanosensor based on our discovery of a “hyper-polarizing lipophilic phase” in which dinonylnaphthalenesulfonate (DNNS^−^) polarizes a solvatochromic dye much more than even an aqueous environment. We have found that the apparent polarity of the organic phase is only modulated when DNNS^−^ binds to large polyions such as protamine, unlike singly charged ions that lack the cooperative binding required to cause a significant shift in the distribution of the polarizing DNNS^−^ ions. Our new sensing mechanism allows solvatochromic signal transduction without the transducer undergoing ion exchange. The result is significantly improved sensitivity and selectivity, enabling for the first time the quantification of protamine and heparin in human plasma using optical nanosensors that correlates with the current gold standard analysis method, the anti-Xa factor assay.

## Abstract 2

Novel optical nanosensors for the selective detection of the polycationic protamine based on solvatochromic signal change allow one to detect heparin in plasma.

## Graphical abstract

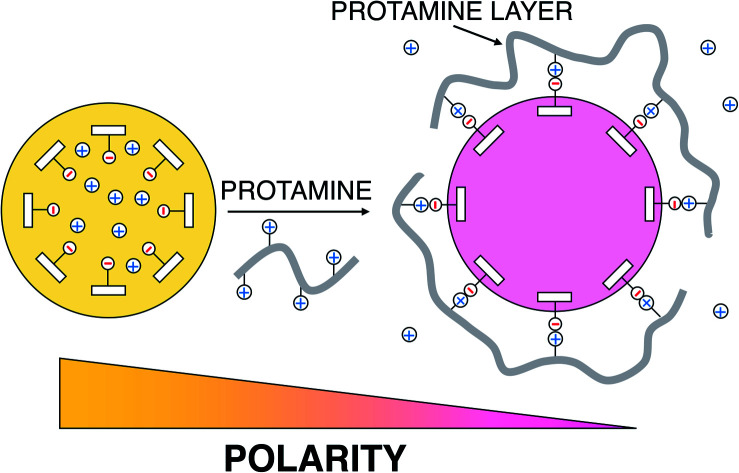

## Introduction

Ion-selective electrodes (ISEs) are among the most robust sensors for the analysis of electrolytes in blood.^1^ Their use for the quantification of polyions in blood has also been explored since the early 1990s particularly due to the demand for establishing a reliable analysis method of heparin, a naturally occurring polyanionic glycosaminoglycan used as an anticoagulant to prevent thrombosis during and after surgery,^2^ and protamine, an arginine-rich polycationic protein generally used to neutralize heparin through polyelectrolyte binding.^2^ This exploration was followed by research on the response mechanism of such polyion-selective electrodes.^3–7^ This mechanism has been of particular interest partially due to the apparent lack of selectivity of the ionophore employed with the sensors relying more on the properties of polyions. A significant amount of research has since been carried out to ascertain whether polyions adhere to the electrode membrane surface or are extracted into the membrane bulk. Early in the development of polyion-selective electrodes, a response theory was proposed based solely on bulk extraction principles, which was found to describe the experimental behavior adequately, indicating that the bulk extraction of polyions may be the dominant mechanism.^3^ However, optical microscopy carried out by Meyerhoff and coworkers suggested significant surface accumulation of protamine.^8^ This was followed by ion transfer voltammetric measurements by Amemiya and coworkers concluding that both adhesion and bulk extraction processes take place in parallel,^9–11^ later confirmed by the group of Meyerhoff.^12^

Following the developments with ISEs, ion-selective optical sensors have emerged as powerful variants of ISEs that allow for an optical readout of ionic species, including polyions.^13–15^ Early examples of optical protamine and heparin sensors used cast sensing films and showed promising performance in diluted serum.^16,17^ Following this work, membrane and particle type sensors that rely on an ion exchange between the target ion and an optical reporter were developed further.^18–22^ Emulsified (nanoparticle-type) optical sensors have been of particular interest as they allow assay miniaturization (to the cellular scale^23,24^), integration of automated liquid handling, and significantly reduced equilibrium times. Unfortunately, nanoparticle-based polyion sensors for reliable measurement in blood, plasma and serum have not yet been successful. For example, Xie and coworkers have reported that the signal of protamine sensors containing dinonylnaphthalenesulfonate (DNNS^−^), a commonly used protamine receptor, was found to completely saturate upon addition of serum to sensors, pointing to important matrix effects^25^ and the need for extreme dilution of serum samples.^26^

In this work protamine and heparin were chosen as model target polyions as convention, and we describe protamine-selective nanoscale optodes employing a fundamentally novel sensing mechanism for assaying heparin in human plasma samples by protamine titration. The research first explores an ion exchange-based assay involving capillary coated sensing films, the basis of which was previously described for the detection of potassium in human serum.^27^

Following this work, we will describe our finding of a “hyper-polarizing organic phase” that forms the basis of a new sensing mechanism. This hyper-polarizing organic phase was produced by adding a large excess of DNNS^−^ : DOS at a 2.8 : 1 weight ratio, thereby DNNS^−^ acts as a solvent rather than a solute. The electron acceptor moiety of solvatochromic dyes strongly interacts with the sulfonate group of DNNS^−^, producing a stronger bathochromic shift than even that of an aqueous environment in the organic phase.

Upon creating nanosensors with a hyper-polarizing organic phase, we have found that only relatively large molecules or proteins such as protamine can disrupt the DNNS^−^/dye interaction resulting in a large hypsochromic shift, which fascinatingly was not induced at all by singly charged species including arginine, the main repeating amino acid moiety of protamine. This charge/size cutoff resulted in significantly better selectivity than any previously reported optical nanosensor for polyions relying on ion exchange of sensing components. This feature of our new nanosensor is likely due to the differences in cooperative binding between large and small molecules, which will be discussed in more detail in the Discussion.

Hence, this nanosensor based on a hyper-polarizing organic phase has two fundamental advantages over previously reported sensors: sensitivity due to the larger solvatochromic shift and selectivity due to the size-filtering effect. This trend was seen with two different nanoparticle formulations containing the solvatochromic dyes X3^+^ and SD017. The improved performance allowed both sensors to quantify protamine in concentrated serum. Due to superior optical properties, the X3-based sensor was then taken forward for quantifying protamine in plasma for the first time using an optical nanosensor. The success of protamine quantification in plasma ultimately allowed for the detection of heparin in plasma samples with the X3^+^-based nanoparticle sensor by protamine titration. This was achieved in the narrow concentration range of 0–0.8 U mL^−1^, which is of high demand for heparin level monitoring during heparin neutralization with protamine as a protamine overdose poses a lethal risk and for the treatment of thrombosis patients with heparin which should be kept at around 0.5 U mL^−1^ in blood. The performance was evaluated with clinical samples and cross-correlated with the anti-Xa assay, the gold standard routinely used in hospitals.

## Results and discussion

### Capillary-based optodes

Capillary-based ion-selective membrane sensors were previously shown to exhibit promising performance for the detection of potassium in serum.^27^ An analogous system for the measurement of protamine was explored, using the positively charged dye Malachite Green (MG^+^) as the counter ion of the protamine recognition unit dinonylnaphthalene sulfonate, DNNS^−^. Upon exposure to a protamine-containing sample, protamine is exchanged for MG^+^ (see Fig. S1a†). The resulting solution absorbance then serves as the analytical signal, which works as expected in buffer samples (see Fig. S2†). However, Fig. S1b† shows that increasing concentrations of protamine in spiked serum only give an absorbance increase above 10 μM protamine. A significant concentration of MG^+^ is already displaced by other serum components as evidenced by the large background in the absence of added protamine.

As shown in Fig. S1c,† heparin concentration in pooled serum was quantitatively analyzed by preloading the samples with 15 μM protamine. Unfortunately, the error bars of the calibration curve were too large for an accurate quantification within this range. This is also reflected by the poor correlation obtained for clinical patient plasma samples between the capillary sensor and the anti-factor Xa method used in hospitals as the gold standard method, see Fig. S1d.†

### Solvatochromic optode emulsion

To overcome the release of the dye from the sensing film in the absence of protamine, a more hydrophobic solvatochromic dye, SD017, containing a lipophilic side chain was explored. This class of dye was previously shown to localize at the sensor/solution interface rather than undergoing bulk solution exchange, which was explained by its hydrophobicity.^28^ SD017 was selected for use as a solvatochromic dye owing to its high lipophilicity due in part to its large aliphatic tail and additionally the fluorine moieties present on the charged head group, increasing the hydrophobicity further while maintaining accessibility for solvatochromic effects. To drastically increase the achievable sensor surface area it was decided to explore emulsified optodes, in analogy to previous work.^15^

**Fig. 1:**
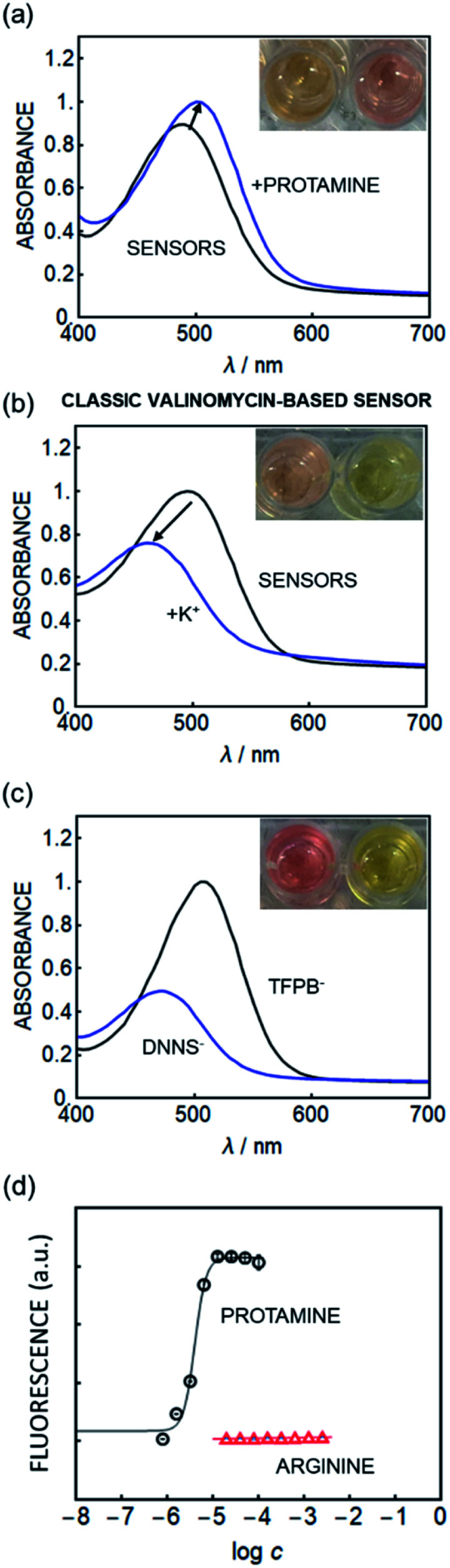
Absorption spectra of (a) the new nanosensor in the SD017-based nanoparticle sensor (which was primarily used for fluorescence readout) in the presence and absence of protamine, suggesting a polarity decrease with increasing protamine concentration. (b) Classical valinomycin-based K^+^ sensor utilizing the same hydrophobic solvatochromic dye in the presence and absence of K^+^ analogous to that reported earlier, showing opposite behavior.^28^ (c) Spectra of SD017 in DOS with either TFPB^−^ or DNNS^−^ as a counter ion, suggesting that protamine removes DNNS^−^ in (a). (d) Fluorescence response of the new nanosensors containing SD017 (510 nm excitation, 610 nm emission) to protamine (black circles) and arginine (red triangles), indicating excellent selectivity (*n* = 3, ±SD).

Surprisingly, optode emulsions containing the hydrophobic solvatochromic dye exhibited a spectroscopic behavior opposite to that expected based on an ion-exchange mechanism (Fig. 1a). To compare, Fig. 1b shows the same dye in a potassium-responsive system containing valinomycin. The extraction of potassium displaces the dye from the nanosensor bulk to the interface by ion-exchange, increasing dye polarity and giving the expected hypsochromic shift. In contrast, Fig. 1a shows a bathochromic shift in the presence of protamine for the DNNS^−^-based emulsion.

To understand the origin of this transition the absorbance of this solvatochromic dye in the bulk solvent DOS (without the aqueous phase) using either DNNS^−^ or TFPB^−^ as a counter ion was measured, see Fig. 1c. The presence of DNNS^−^ alters the dye's polarity in a manner analogous to that observed in the absence of protamine as shown in Fig. 1a. The bulk solution experiments suggest that the optical response occurs within the particle bulk and is given by a local polarity change involving the availability of DNNS^−^.

### Optical nanosensors based on solvatochromicity

**Fig. 2:**
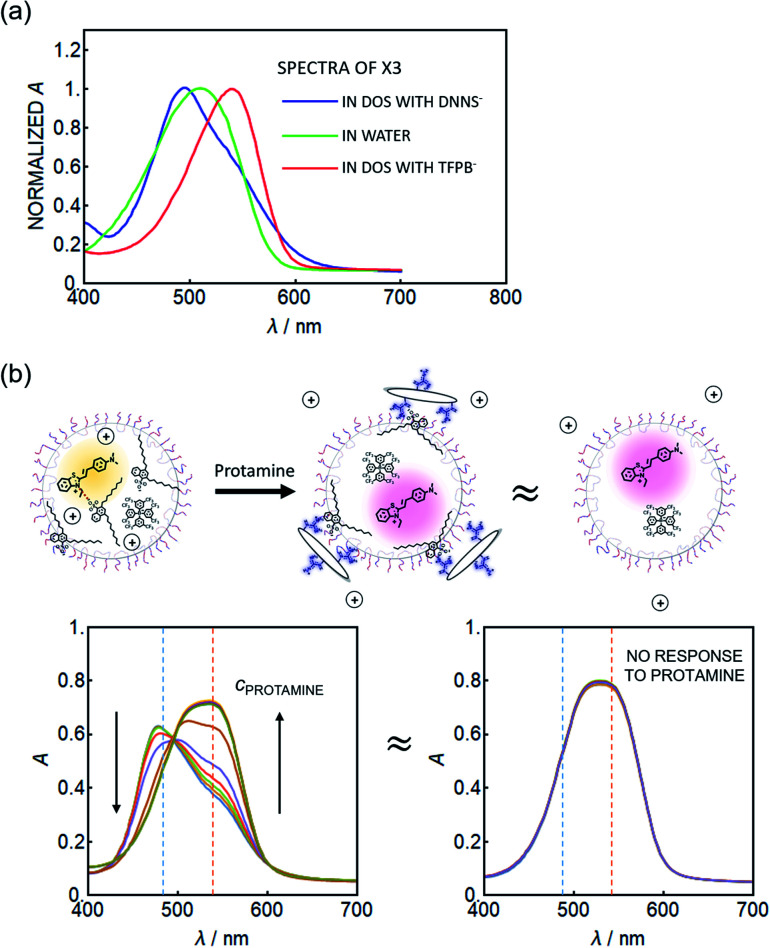
(a) Normalized absorption spectra of X3 in the bulk solution of DOS containing DNNS^−^ (blue) or TFPB^−^ (red), and of X3 in water (green). (b) Emulsified sensor responses to protamine. Left: sensors containing DNNS^−^, TFPB^−^ and X3. Right: the same conditions but without DNNS^−^, giving no response. The absorption spectra of the sensor in the presence of protamine (left spectra at high protamine concentration) are “almost equal” to those of the nanoparticles containing only X3^+^ and TFPB^−^ (right figure), indicating that the internal composition is likely also similar or equivalent.

Because the dye SD017 gave relatively small spectral changes, another solvatochromic dye, X3^+^, was explored which exhibits a greater solvatochromic absorbance shift. This dye is also more hydrophilic. Fig. 2a shows the normalized absorption spectra of the solvatochromic dye X3^+^ in water (dark blue trace), showing a peak maximum around 505 nm. In an organic solvent (DOS) containing a lipophilic, non-coordinating cation-exchanger, TFPB^−^, the absorbance peak red shifts to about 545 nm. The four sterically bulky phenylborate groups of TFPB^−^ are known to symmetrically distribute the negative charge.^23^ If this bulky ion-exchanger is replaced by DNNS^−^, used to recognize protamine, the absorbance is found to blue shift to a maximum around 495 nm (see Fig. 2a). This is attributed to the highly charged sulfonate group of the DNNS^−^ ion pairing with the X3^+^ benzothiazolium group. The large blue shift suggests a larger polarization of the dye than induced by water as solvent, which is a finding of particular interest. In this “hyper-polarizing organic phase”, one may recognize DNNS^−^ as a solvent rather than a solute considering the high DNNS^−^ : DOS ratio (1 : 1). A molar ratio of DNNS^−^ : DOS of 1 : 1 was used here, in contrast to 2.8 : 1 in the actual nanosensor, because of the high absorbance of DNNS^−^ which placed spectrophotometric limits on this experiment. For this reason a larger blue shift (peak at 460–480 nm) was observed for the emulsified nanosensor particles.

Polycationic protamine is expected to interact with DNNS^−^, disrupting the association with X3^+^ and hence resulting in a shift of the peak maximum to longer wavelengths. The nanoparticle sensor for protamine detection contained the solvatochromic dye X3^+^ as an optical transducer along with the lipophilic cation-exchanger TFPB^−^ as a counter ion to stabilize it in the emulsified organic sensing phase. The combination of just these two reagents did not result in a response to protamine, see Fig. 2b (right). When the sensing phase additionally contained a molar excess of protamine-selective anion-exchanger DNNS^−^, increasing protamine levels continuously decreased the initial peak at 480 nm, forming a new red shifted peak around 545 nm. This suggests that the initial interaction between the solvatochromic dye X3^+^ and DNNS^−^ is continuously disrupted by increasing levels of protamine. When their interaction is completely disrupted, the spectrum of the nanosensor (see Fig. 2b, high *c*_protamine_) looks very similar to that of the combination of X3^+^ and TFPB^−^ (see Fig. 2b, right).

**Scheme 1:**
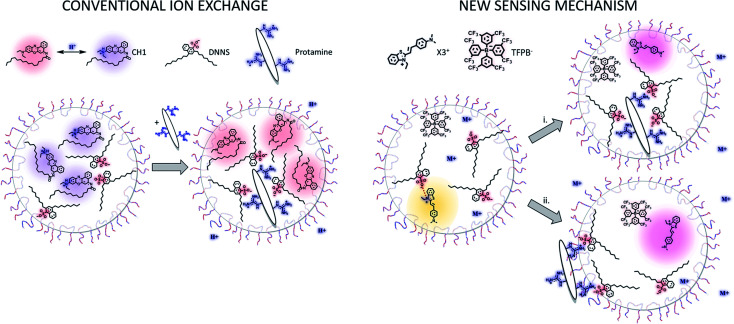
Comparison of conventional ion exchange based on a H^+^-chromoionophore (CH1) (left) with the novel polarity indicating sensing mechanism (right) in F-127-emulsified particles with the plasticizer DOS as a sensing matrix. In route (i) protamine extracts into the sensor bulk phase, while in route (ii) it adsorbs onto the surface by drawing DNNS^−^ away from the sensor bulk. In both cases, a hydrophilic counterion is co-extracted to maintain charge balance.

This type of optode composition differs from that used in conventional bulk optodes where a pH-responsive chromoionophore serves as a reporter dye (Scheme 1, left). In that case, protamine is extracted into the organic sensing phase to interact with DNNS^−^, expelling hydrogen ions to maintain electroneutrality and deprotonating the pH-responsive dye in the process. Because in the new approach the solvatochromic dye does not function as a pH indicator, the sensing mechanism is different.

As X3^+^ is more hydrophilic than SD017 we were mindful of the possibility of dye exchange into the aqueous phase. However, dialysis experiments with the emulsified sensing phases (see Fig. S3†) indicated negligible dye leakage from the nanoparticles both in the absence and presence of protamine. This suggests that ion exchange of X3^+^ with protamine can be excluded. Moreover, the observed red shift upon introduction of protamine suggests that the solvatochromic dye experiences an environment of lower polarity, which is not compatible with classical ion-exchange into aqueous solution. Rather, the evidence points to X3^+^ being localized in the bulk organic sensing phase after protamine interaction with DNNS^−^.

Based on this, two different mechanisms are proposed (see Scheme 1 right). In the more classical scenario (route (i)), sensing is by bulk ion-exchange. Protamine enters the organic sensing phase and expels the inorganic counter cation of DNNS^−^. The interaction between protamine and DNNS^−^ breaks the ion pair with X3^+^, which remains in the sensing phase as a counter cation of TFPB^−^. An alternative explanation of the data suggests that protamine is not appreciably extracted but instead adsorbs onto the sensing particle surface. For this, DNNS^−^ is removed from the sensing phase together with a counter cation to the interface, again breaking the interaction between X3^+^ and DNNS^−^ to give rise to the observed red shift (route (ii)). The main difference between the two mechanisms is where protamine settles. Both are in principle possible because protamine and other polyions have been reported to extract to the organic sensing phase and also to be adsorbed at the interface, as evidenced by electrochemical and optical experiments (see the Introduction for more details). The observed zeta potential changes in the absence and presence of protamine are shown in Table S1.† Unfortunately, the changes may be too slight to fully support one mechanism over the other; however, we did observe the expected shift from a negative to positive zeta potential following the addition of protamine.

The new sensing mode is found to depend strongly on the DNNS^−^ concentration in the sensing phase, which was here experimentally changed through the plasticizer concentration. Fig. S4† shows the sensor response to protamine with different concentrations of DOS. The solvatochromic signal change becomes less pronounced with a higher dilution of active ingredients, which narrows the dynamic range.

The sensor operates in an exhaustive manner in which nearly all protamine molecules in the sample solution are extracted into (or onto) the sensor nanoparticles. Fig. S5† shows the protamine response of Fig. 1a but plotted with non-logarithmic concentrations (blue circle) in comparison to nanosensors having a higher concentration of DNNS^−^ (yellow triangle). The response of the sensor with a lower DNNS^−^ concentration is clearly linear in nature, indicating exhaustive operation. Interestingly, the response for the high DNNS^−^ concentration becomes weaker at low protamine concentration. This may be caused by the excessive DNNS^−^ already decorating the solution–particle interface in the absence of protamine, which will no longer modulate inner particle polarity when protamine starts being present. Although DNNS^−^/protamine complexation is exhaustive, the signal transduction range can be tuned in this manner. Because the sensing mechanism is exhaustive,^20^ the sensitivity of the system can be further modulated by altering the total particle concentration.

**Fig. 3:**
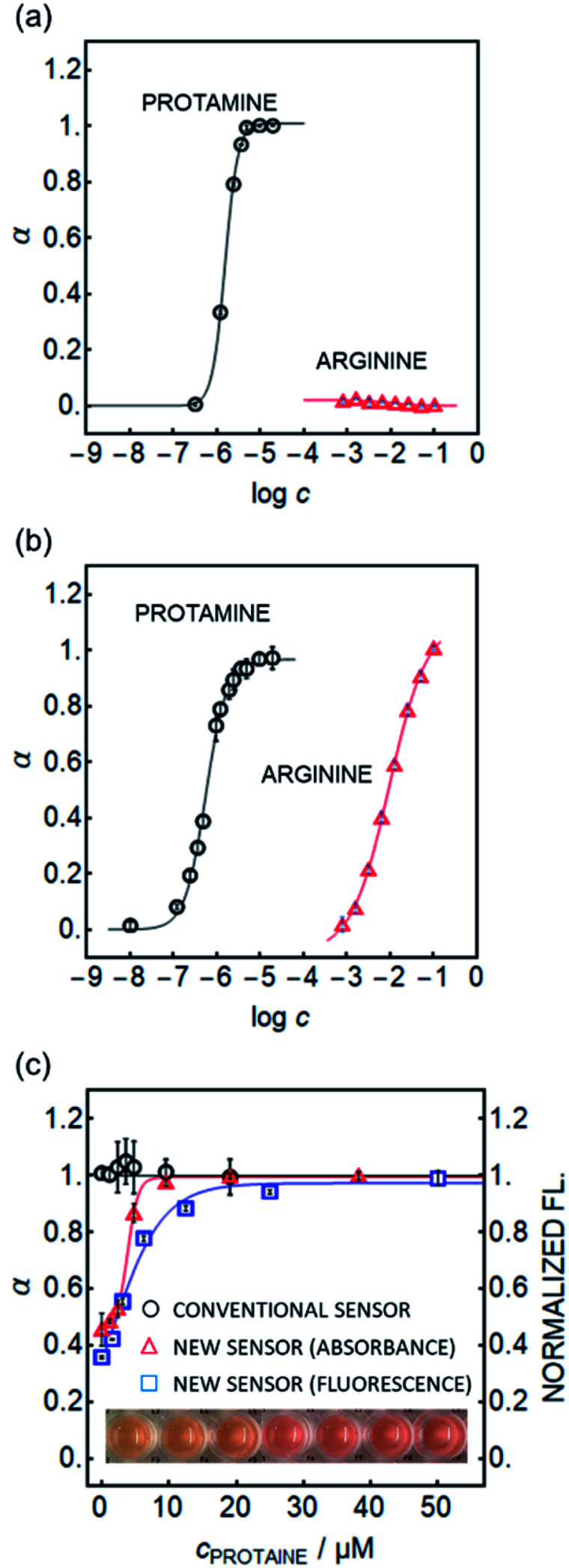
Response to protamine (circles) and arginine (triangles) of (a) the new nanosensor containing DOS, DNNSH, X3^+^ and KTFPB and (b) of the conventional ion exchange sensor containing CH1 and DNNS^−^ (*n* = 3, ±SD). Alpha on the *y*-axis is the reaction ratio, taking fully polar signal as 0 and fully non-polar signal as 1. (c) Response of the conventional CH1-based sensor (black circles) and the new nanosensor based on X3 (absorbance, shown as triangles), and based on SD017 (fluorescence; squares) in pooled plasma (*n* = 3, ±SD). Normalized fluorescence intensity was placed on the right *y*-axis for the plots of fluorescence readout. The pictures below show the color change of the new nanosensor in plasma containing increasing protamine concentrations from left to right.

A selectivity test using arginine (Arg), the main repeating amino acid of protamine, suggests that the nanosensors developed here function in a different manner to conventional sensors. As shown in Fig. 3a and b (absorbance readout) and Fig. 1d (fluorescence readout), the new sensor shows no detectable response even to 100 mM arginine, while the conventional ion exchange type sensor (Scheme 1 left) shows significant interference.

This improved selectivity was also observed for other potentially interfering species, such as Ca^2+^, Mg^2+^ and Na^+^ (see Fig. S6†). This finding might more easily support route (ii), as the polyionic species interact with the DNNS^−^ species drawn to the interface in analogy to the strong adsorption of the polyelectrolyte layer onto charged surfaces. This may result in an effective discrimination of ionic species of lower valency that lack this cooperative character and rather extract into the bulk particle phase as DNNS^−^ counterions. Because the hydrophilic protamine is not required to extract into the organic phase to achieve interaction, the energy barrier to pull out DNNS^−^ from the bulk particle phase to the outer surface may be lower than that for arginine and inorganic ions.

Owing to the promising selectivity, both absorbance and fluorescence-based nanosensors were found to be responsive to increasing protamine concentration in serum with higher sensitivity (see Fig. 3c) than the capillary sensor (see Fig. S1b†). This is in contrast to ion-exchange type optodes where no apparent response with emulsified optodes was observed, see Fig. 3c.^20^

Fig. 3c shows that the response in serum before the addition of protamine shows an elevated baseline. One possibility for this would be the presence of small cationic species in serum. However, the addition of 2.0 mM arginine to displace any other small cations from the sensing phase before protamine addition did not change the calibration curve. Indeed, Fig. S7† shows that the nanosensor signals to protamine in buffer are the same when Na^+^ or arginine is used as the counter ion. Another possible interference might stem from phospholipids that may fuse with the sensing emulsion to disrupt its function. As observed in Fig. S8,† however, the addition of phospholipid micelles^29^ was not found to influence the optode response and this interference may also be excluded.

**Fig. 4:**
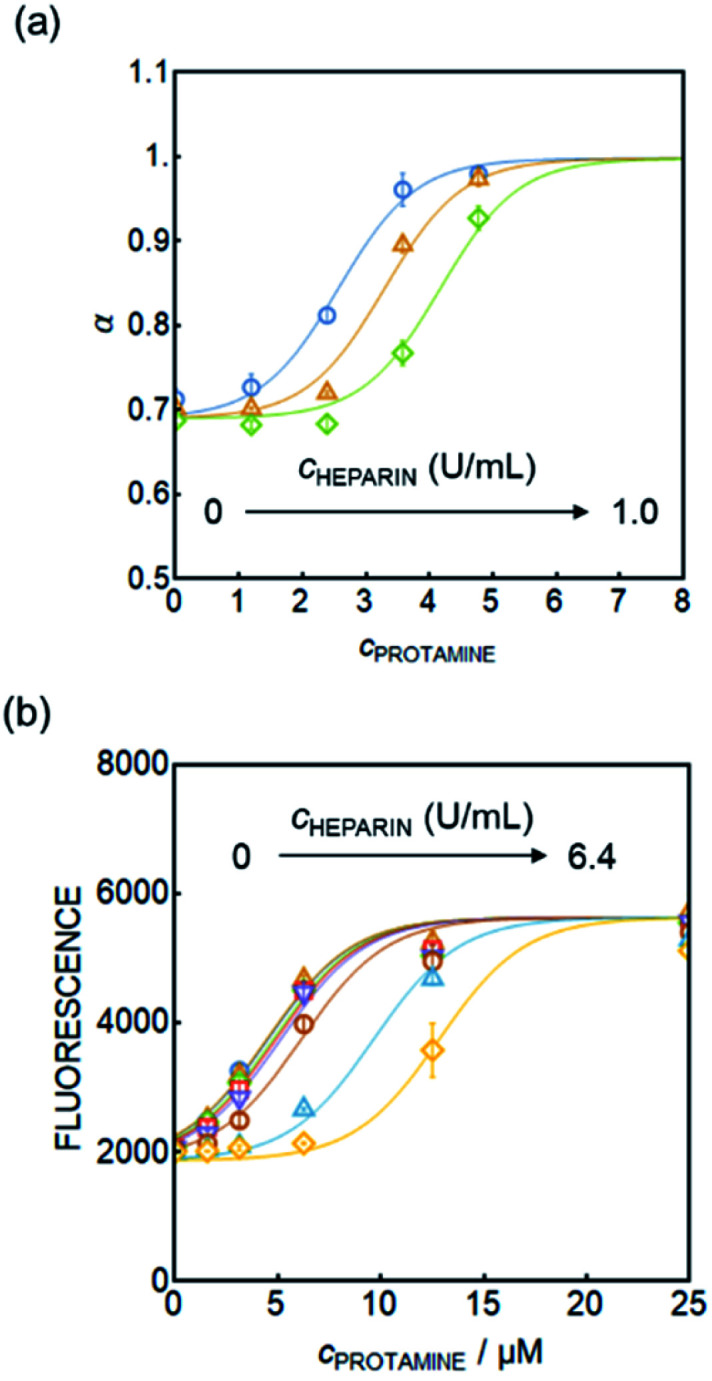
Protamine response curve of (a) the new nanosensor (absorbance readout) in pooled plasma containing 0, 0.5, and 1.0 U mL^−1^ heparin and (b) the curve of the new nanosensor (fluorescence readout) in pooled serum containing 0, 0.1, 0.2, 0.4, 0.8, 1.6, 3.2, and 6.4 U mL^−1^ heparin (*n* = 3, ±SD).

At this stage it is assumed that the major interference originates from peptides containing protamine-like moieties and lipophilic proteins in serum that may alter the hydrophobicity of the particles, but more work is required to further elucidate this point. Despite the elevated baseline, the protamine calibration curves in plasma were found to shift to higher concentrations upon addition of heparin for sensors based on both absorbance and fluorescence readout, see Fig. 4a and b. This demonstrates their potential to measure heparin reliably in physiological samples.

### Heparin quantification in human plasma samples

The nanosensors were used to quantify low heparin concentrations in plasma samples (0–0.8 U mL^−1^) sourced from the University Hospital of Geneva (HUG) for which cross-correlation data with anti-Xa assay were available. These heparin levels are significantly lower and analytically more challenging than the range reported earlier with potentiometric heparin sensors (1–10 U mL^−1^heparin). Consistent with expectations, the nanosensors were confirmed to respond independently of pH in a wide range (pH 5–9), see Fig. S9,† which is important since plasma pH may vary slightly between patients. Eventually, the nanosensors may undergo hydrogen ion interference upon neutralization of the DNNS^−^ sulfonate group, but this would be outside the physiological range.

**Fig. 5:**
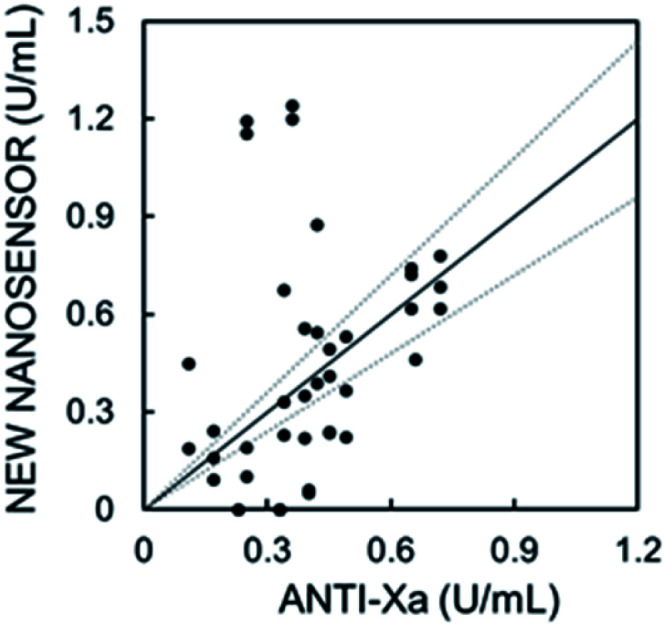
Correlation plot of measured heparin concentration using the new nanosensor with absorbance readout. Spearman correlation with a one-tailed *t*-test gives *r* = 0.44 and *p* = 0.04, indicating better correlation than for the capillary sensor (Fig. S1d†).

Fig. 5 shows the cross-correlation with 17 patient samples. Plasma samples containing heparin were doped with different, known concentrations of protamine, followed by the addition of nanosensor emulsion solution. The protamine concentration required to produce a 50% signal change was taken from a sigmoidal fit and was used to determine the heparin concentration. The curves in Fig. 5a and S10† which were obtained in pooled plasma were used as the standard response for calibration. The fabrication procedure in both cases is the same, and slight differences are attributed to batch variation. Protamine response curves for each patient sample are shown in Fig. S11 and S12.† Fig. S10† was used for patients in Fig. S11† and 4a for Fig. S12.†

Considering the previously reported cross-correlations of various newly developed sensors and devices specifically towards the anti-Xa assay,^5,30–33^ having *r* = 0.22–0.89 at 5 times higher concentration ranges than measured here, the data from the nanosensors correlate relatively well to the current gold standard method for heparin quantification in hospitals. A Spearman correlation with a one-tailed *t*-test for the data in Fig. 5 gives a correlation of *r* = 0.44 and *p* = 0.04 indicating a moderate correlation of statistical significance, which is a major improvement from the capillary-based sensor shown in Fig. S1d,† particularly for such a low heparin level (0–0.8 U mL^−1^). We assume that the error observed here arose from the matrix effect that differs from patient to patient. Our new sensors are also capable of heparin level analysis at higher concentration relevant to open heart surgery as demonstrated in Fig. 4b, and the matrix effects are expected to become negligible as higher heparin concentrations are measured.

As shown above, the new sensing mechanism has enabled protamine and heparin analysis in concentrated serum and plasma samples. Moreover, the application of our sensor is not limited to only these two polyionic species. For example, α-poly-l-lysine (polylysine) can also be measured using our new sensor as shown in Fig. S15.† The success with polylysine suggests broad applicability of our mechanism for the measurement of a variety of polyionic species. We assume that the polarising ionophore (DNNS) could also be replaced by other molecules with similar properties: highly lipophilic compounds with groups possessing highly localised charge such as carboxylates and phosphates. Indeed, we expect that the polarising ionophore does not necessarily have to be an anion either. Fig. S16† shows the spectral change of merocyanine 540, a solvatochromic dye with an overall negative charge, and tridodecylmethylammonium (TDMA^+^), a cationic ligand used to induce polarisation. Based on the above results, we believe that the described hyperpolarization-based sensor possesses broad potential for the measurement of other polyions and will facilitate nanoscale polyion sensing in biological samples.

## Conclusions

We have developed a new protamine/heparin sensor in which DNNS^−^, a receptor for protamine, dramatically alters the internal particle polarity in the absence and presence of protamine, allowing for a solvatochromic signal transduction. Although the detailed mechanism is not yet entirely clear, experimental data suggested two possible mechanisms involving either the extraction of protamine into the sensing phase or its binding to the sensing particle surface. The new nanosensor showed an excellent improvement in selectivity compared to conventional ion exchange type sensors, which allowed protamine quantification in plasma and serum. Notably, the new sensor showed complete absence of interference from arginine, the major amino acid component of protamine, which is in contrast to conventional sensors. The promising performance of the new sensor strategy was confirmed in 17 patient samples by comparing it to the anti-Xa assay used as the gold standard. We are now developing new device strategies based on this chemistry to further reduce biological fouling and enhance heparin quantification capabilities.

We believe that our sensing mechanism can be applied to other combinations of receptors and polyions. In our laboratory, nanosensors for biologically relevant polyanions such as DNA, RNA and phosphorylated proteins are under development. In addition, this nanosensor should in principle be able to operate *in situ* for optical sensing of polyion polymerization processes due to the size effect in which hypsochromic effects are observed as arginine polymerization proceeds.

The application of these newly developed sensors to whole blood may be achieved by embedding these sensors in a gel, in analogy to the recent work by Xie's group.^34^ This approach would exclude blood cells from the gel-based sensing phase and allow for the determination of protamine/heparin in whole blood.

## Author contributions

Conceptualization, Y. S. and E. B.; experiments, Y. S., R. N. and K. J. R.; writing – original draft, Y. S. and K. J. R.; writing – review and editing, Y. S., K. J. R. and E. B.; supervision, E. B.

## Conflicts of interest

There are no conflicts to declare.

## Supplementary Material

SC-012-D1SC04930E-s001
