# Supplementary material for: Protamine/heparin optical nanosensors based on solvatochromism
Source: Chem Sci. 2021 Nov 15;12(47):15596–602. doi: 10.1039/d1sc04930e (PMC8653997; doi:10.1039/d1sc04930e)
Supplement: SC-012-D1SC04930E-s001 [file SC-012-D1SC04930E-s001.pdf]

Electronic Supplementary Material (ESI) for Chemical Science.
This journal is © The Royal Society of Chemistry 2021

Supporting Information

Protamine/Heparin Optical Nanosensors based on Solvatochromism

Yoshiki Soda+, Kye J. Robinson+, Robin Nussbaum, and Eric Bakker*

Department of Inorganic, Analytical Chemistry
University of Geneva
Quai Ernest-Ansermet 30, 1211 Geneva, Switzerland
E-mail: Yoshiki.Soda@unige.ch, Kye.Robinson@unige.ch, Eric.Bakker@unige.ch

+Y. S. and K. J. R. contributed equally to this this work.

Abstract: Optical nanosensors for the detection of polyions, including protamine and heparin, have to date relied upon ion-exchange reactions involving an analyte and an optical transducer. Unfortunately, due to the limited selectivity of the available ionophores for polyions, this mechanism has suffered from severe interference in complex sample matrices. To date no optical polyion nanosensors have demonstrated acceptable performance in serum, plasma or blood. Herein we describe a new type of nanosensor based on our discovery of a "hyper-polarizing lipophilic phase" in which dinonylnaphthalenesulfonate (DNNS-) polarizes a solvatochromic dye much more than even an aqueous environment. We have found that the apparent polarity of the organic phase is only modulated when DNNS- binds to large polyions such as protamine, unlike singly charged ions that lack the cooperative binding required to cause a significant shift in the distribution of the polarizing DNNS- ions. Our new sensing mechanism allows solvatochromic signal transduction without the transducer undergoing ion exchange. The result is significantly improved sensitivity and selectivity, enabling for the first time the quantification of protamine and heparin in human plasma using optical nanosensors that correlates with the current gold standard analysis method, the anti-Xa factor assay.

Table of Contents

Experimental Procedures 2
Additional Results 6
References 16
Author Contributions 16

Experimental Procedures

Materials and Instruments.

Dioctyl sebacate (DOS), Potassium tetrakis3,5-bis(trifluoromethyl)phenylborate (KTFPB) Selectophore^{TM} grade, sodium tetrakis3,5-bis(trifluoromethyl)phenylborate (NaTFPB) Selectophore^{TM} grade, Potassium ionophore I Selectophore^{TM} grade, chromoionophore I (CH1) Selectophore^{TM} grade, Pluronic F127, tetrahydrofuran (THF) Selectophore^{TM} grade, 2-Nitrophenyl octyl ether (NPOE) Selectophore^{TM} grade, poly-L-lysine hydrochloride (Mw: 15000-30000), Tridodecylmethylammonium (TDMA), tetradodecylammonium (TDA), Merocyanine 540, boronic acid, citric acid, monosodium phosphate, sodium hydroxide (NaOH) and lipid standard triglyceride mixtures were purchased from MilliporeSigma (Burlington, USA). Dinonylnaphthalenesulfonic acid (DNNSH) solution was sourced from Santa Cruz Biotechnology (California, USA). Cholesterol was purchased from Chemodex (St. Gallen, Switzerland). 1,2-Dipalmitoyl-sn-glycero-3-phosphocholine was sourced from Tokyo Chemical Industry (Tokyo, Japan). Pooled plasma sample was purchased from Biowest (Nuaillé, France) while Hôpitaux universitaires de Genève (Geneva University Hospitals, HUG) provided plasma sampled from 17 patients. Citrated tubes from Becton Dickinson (New Jersey, USA) were used to collect blood from patients. Absorbance and fluorescence measurement was performed by TECAN Infinite M Nano$^+$ microplate reader (Tecan, Zürich, Switzerland) with 96 and 384 well plate sourced from Eppendorf (Hamburg, Germany) and Greiner AG (Kremsmünster, Austria) respectively. Zeta potential measurements were performed by Zetasizer Nano and folded capillary cuvette (Malvern Panalytical, Malvern, UK). 827 pH lab (Metrohm, Herisau, Switzerland) was used to measure pH in buffer preparation. The anti-Xa assay was performed on an Atellica COAG 360 instrument (Siemens, Germany) using the Biophen Heparin LRT reagent (Hyphen Biomed, France). X3 solvatochromic dye was synthesized as previously reported.[1] SD017 solvatochromic dye was synthesized using a method adapted from Wang *et al.* see supporting information.[2]

Fabrication of capillary sensor and measurement in plasma.

1.4 mg of DNNSH, 0.68 mg of malachite green (MG), 2.1 mg of poly(vinyl chloride) and 4.2 mg of DOS were dissolved in 2 mL THF. Capillary optodes were fabricated as reported previously.[3] Pooled and patient plasma samples were doped with 15 µM protamine. 50 µL of the protamine-doped plasma samples were aspirated into the capillary and incubated for 5 min. The sample was then discharged into a cuvette containing 1 mL deionized water. Absorbance of the cuvette was then measured using a SPECTRO 250 PLUS UV/VIS spectrophotometer (Analyticjena, Jena, Germany).

Preparation of 500 mM universal buffer adjusted at pH 7.4.

A stock solution containing 1 M boronic acid, 1 M citric acid and 1 M monosodium phosphate was prepared. The pH of this solution was tracked by a commercial 827 pH lab pH meter and adjusted to 7.4 by adding 5 M NaOH. Then, the pH-adjusted buffer solution was diluted to reach a boronic acid, citric acid and monosodium phosphate of 500 mM.

Preparation of the new protamine/heparin nanosensor for absorbance readout.

For fundamental research in buffer, 4.20 or 2.80 mg of DNNSH, 1.00 mg of X3, 1.80 mg of KTFPB and 2 mg of DOS were dissolved in 2 mL of methanol. Note that dye X3$^+$ is has low solubility in THF typically used in optode preparation. This mixture was cast into a beaker containing 50 mL of 0.02 wt % Pluronic® F127 aqueous solution. Methanol was removed by blowing compressed air into the beaker for more than 3 hours at room temperature. Note that some water was lost in this process too. The resulting emulsion had very clear orange color and was not cloudy at all. In turn, this emulsion solution and 1 mL of 500 mM universal buffer (see above for the buffer preparation method) were poured into a 50 mL volumetric flask and water was filled to the etched line to obtain nanosensors adjusted at pH 7.4 with 10 mM universal buffer. When arginine was used as a counter ion before sensing, 5 mL of 42 mM arginine solution was also poured into the volumetric flask before filling up by water.

Nanoparticles containing only TFPB$^-$ and X3 used in Figure 1 were prepared in the same manner but without DNNSH.

For nanosensors used in serum, 2.5 mg of DNNSH, 0.45 mg of X3, 0.81 mg of KTFPB and 1 mg of DOS were dissolved in 2 mL of methanol. Buffered nanosensor solutions containing arginine as a counter ion was prepared in the same manner as above.

Preparation of the new nanosensor for fluorescence readout.

Nanosensors for fluorescence based measurements were produced by combining 8.84 mg of DNNSH, 0.5 mg of SD017, 0.5 mg of NaTFPB and 6.7 mg of NPOE in 3 mL THF. NPOE was used instead of DOS because the florescent signal of SD017 showed drift over time in DOS but was stable in NPOE. 0.4 mL of this cocktail was then transferred into a 10 mL vial containing 4mL of 0.02 wt % F127 in water which was then shaken under a stream of compressed air for one hour using a Scientific Industries Vortex Genie 2 (setting 2). Following this 100 µL of 500 mM universal buffer was added and the solution made up to 5 mL.

Preparation of the classical K^+ fluorescence sensors using SD017.

Potassium nanosensors using SD017 were produced by combining 1.01 mg of K^+ ionophore I, 0.6 mg of SD017, 0.6 mg of NaTFPB and 6.7 mg of DOS in 3 mL THF. 0.4 mL of this cocktail was then transferred into 4mL of 0.02 wt % F127 in water which was then shaken under a stream of compressed air for one hour.

Absorbance readout method.

For measurement in buffer, 100 µL of the buffered nanosensor solution was pipetted into each well of 96 well microplates (UV/VIS flat bottom from Eppendorf) followed by pipetting 100 µL sample solutions (protamine, poly-L-lysine, arginine and lysine). In turn, the well plate was inserted into TECAN Infinite M Nano$^+$ microplate reader. The software "i-control" provided by Tecan was used to initiate absorbance measurements. The same procedure was taken for the pH independency and selectivity investigation.

For measurement in pooled plasma, the auto injection function of the microplate reader was used to automate the analysis. 100 µL of plasma samples containing 0, 1.19, 2.39, 3.58, 4.77, 9.55, 19.09 µM of protamine and 0, 0.3, 0.5, 0.6, 0.9 or 1.0 U/mL heparin were placed in each well of 96 well microplates (UV/VIS flat bottom, Eppendorf). The plate was inserted into the Tecan microplate reader. Buffered nanosensor solution containing 2 mM arginine was placed in the injection module of the microplate reader. As programed in i-control, 100 µL nanosensor solutions were dispensed into all the wells, after 2 min the absorbance at 460 and 530 nm was measured with injection matched delay.

For measurement in plasma from patients, 40 µL of the remaining patient samples from the anti-Xa assay dosed with 0, 1.19, 2.39, 3.58, 4.77, 9.55, 19.09 µM protamine were placed into wells of 384 well microplates (transparent from Greiner). The measurement procedure is same as the pooled serum analysis except that injection volume, here 40 µL. Note that proper plate configuration should be chosen in i-control program.

The program of Tecan microplate reader used in protamine/heparin measurement in patient plasma samples by the newly developed nanosensors is shown below. In this program, nanosensor solution was injected into all the wells holding sample solutions at once and absorbance of each well was measured with an injection-matched delay.

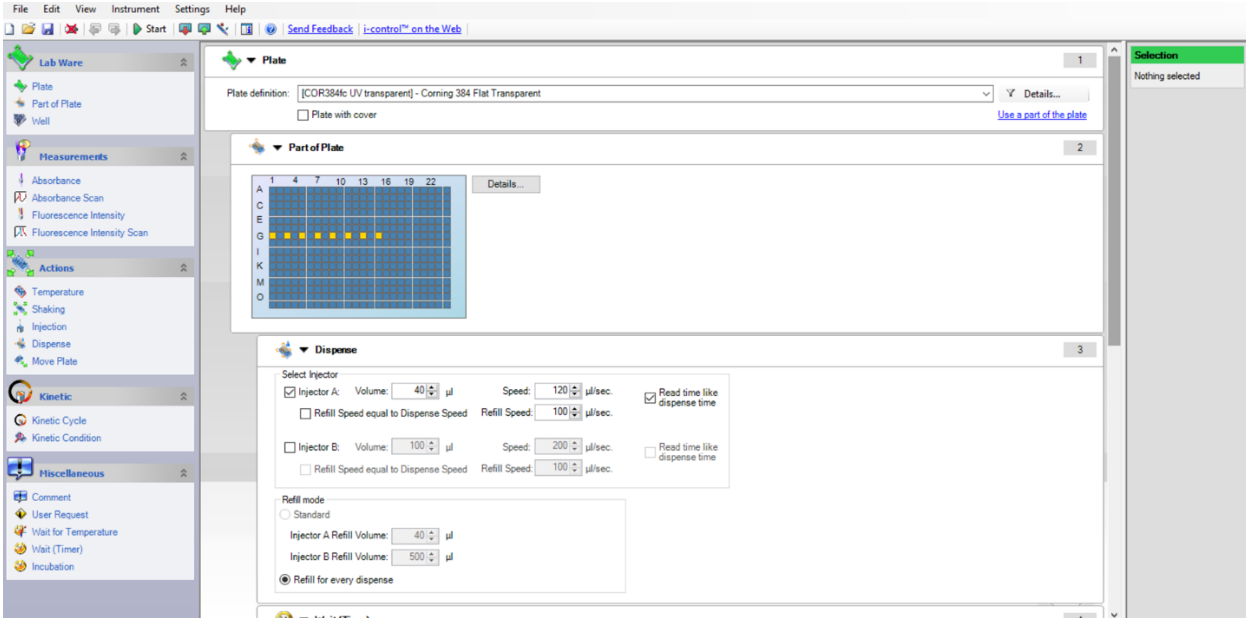

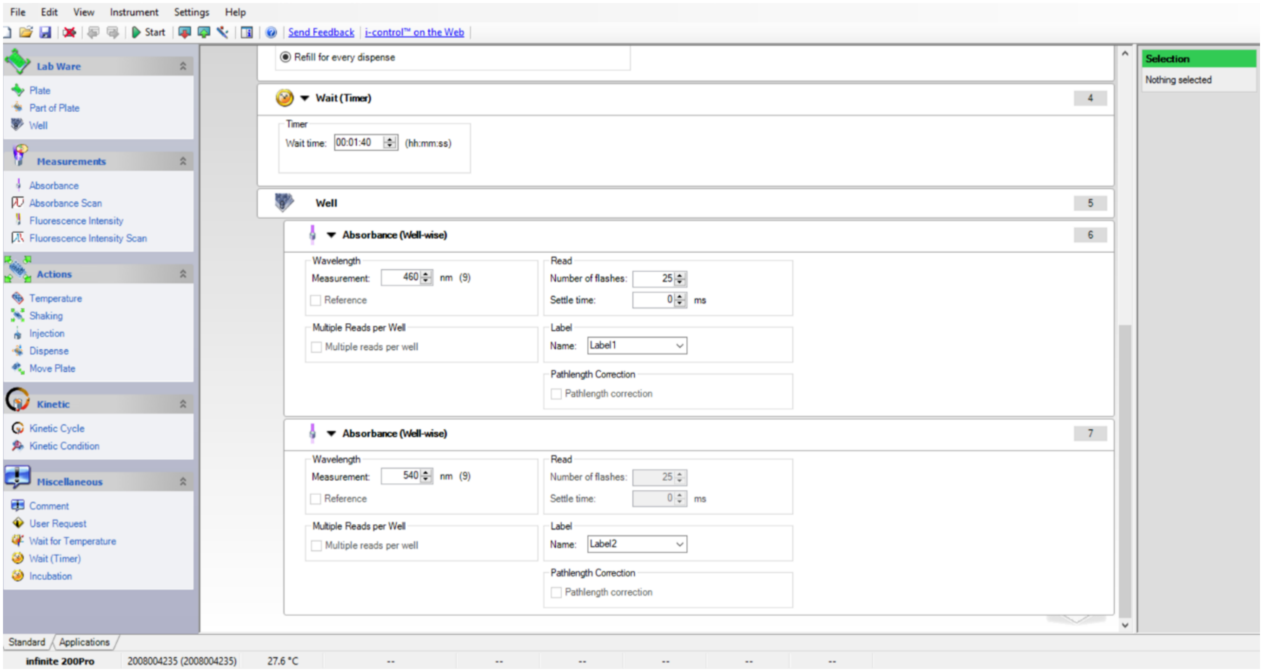

Fluorescence readout method.

For fluorescent measurements in pooled serum, 100 µL of serum was added to 96 well cell imaging microplates (Eppendorf). To this 10 µL of heparin solution was spiked using a multichannel pipette to reach final concentrations of 6.4, 3.2, 1.6, 0.8, 0.4, 0.2, 0.1 and 0 unit/mL. Following this 50 µL of protamine at concentrations ranging from 0.2 mM to 3.125 µM using a 1:2 dilution factor was added to the Serum. Finally 50 µL of fluorescent nanosensors was added and fluorescence measured at 510 nm excitation and 610 nm emission using the Tecan microplate reader after 3 minutes.

Anti-Xa factor measurement on patient plasma samples.

2.7 mL of blood samples were taken from patients following treatment with heparin and stored in citrated tubes containing 0.3 mL of 109 mM sodium citrate solution to prevent blood coagulation. In turn, the blood samples were centrifuged at 2500 G for 10 min within 1 hour of the blood collection. As soon as plasma samples were obtained after removing blood cells by the centrifugation, their heparin levels were measured by the anti-Xa factor method performed by an Atellica COAG 360 instrument.

Preparation of CH1 nanoparticles and measurement method.

1.3 mg of DNNSH, 1.0 mg of chromoionophore I, 10 mg of DOS and 5 mg of F-127 were dissolved into 2mL of tetrahydrofuran. The mixture was injected into 50 mL of deionized water and THF was removed with the compressed air in the same manner as above.

The measurement procedure is the same as that used for the new nanosensor in buffer except absorbance was taken at 530 and 670 nm (see Measurement method for absorbance readout.)

Dialysis test with particles containing $X3^+$ solvatochromic dye.

4.20 mg of DNNSH, 1.00 mg of $X3^+$, 1.80 mg of KTFPB and 2 mg of DOS were dissolved in 2 mL of methanol. This mixture was cast into a beaker containing 50 mL of 0.02 wt % Pluronic® F127 aqueous solution. Methanol was removed by blowing compressed air into the beaker for more than 3 hours at room temperature. In turn, this emulsion solution and 1 mL of 500 mM universal buffer (see above for the buffer preparation method) were poured into a 50 mL volumetric flask and water was filled to the etched line to obtain nanosensors adjusted at pH 7.4 with 10 mM universal buffer. 50 µM protamine solution was added to this nanoparticle sensor solution, which was finally poured into a dialysis bag.

To prepare particles containing only a dye and $TFPB^-$, 1.00 mg of $X3^+$, 1.80 mg of KTFPB and 2 mg of DOS were dissolved in 2 mL of methanol. Nanoparticles are fabricated in the same manner and this solution was poured into a dialysis bag.

Dialysis bags were soaked in a 5 L of deionized water for a day, which was repeated for three times to confirm the absence of dye leakage.

Zeta potential measurement of the new nanosensors in the presence and absence of protamine.

1 mL of the new nanosensors (see above for the preparation method) buffered at pH 7.4 by 10 mM universal buffer was pipetted into folded capillary zeta cells (Malvern Panalytical) containing either 0, 4 or 84 µM protamine. Zeta potential of particles in these cuvettes was measured using a Zetasizer Nano (Malvern Panalytical).

Fouling test with artificial plasma lipid components.

15 mg of lipid standard triglyceride mixture, 17 mg of cholesterol and 22.2 mg of 1,2-dipalmitoyl-sn-glycero-3-phosphocholine were dissolved in 2 mL of THF. The THF mixture was then poured into 10 mL deionized water. Lipid emulsion was obtained after THF was removed in the same manner as above.

100 µL of the lipid emulsion solution was mixed with 100 µL of the nanosensor solution on the 96 well plate and the absorbance was measured in the Tecan microplate reader.

Polarization of Merocyanine 540 (Mc540) by tridodecylmethylammonium ion (TDMA$^+$).

For particles having polarizing nature, 1mg of DOS, 0.51 mg of Merocyanine 540 and 2.17 mg of tridodecylmethylammonium chloride (TDMACl) and 0.64 mg of tetradodecylammonium chloride (TDACl) were dissolved in 2 mL of methanol. For particles of nonpolar nature, the methanol mixture did not contain TDMACl but otherwise had the same composition. Particles were fabricated in the same manner as protamine sensors. The absorption spectrum of these two particle solutions were recorded by TECAN microplate reader.

SD017 Preparation Method

4-Methylpyridine (**1**) 98% and 1,1,1-trifluoro-3-iodopropane (**2**) 98% were purchased from MilliporeSigma (Burlington, USA). **4** was synthesized in the lab previously, as described by Wang *et al.*[2]

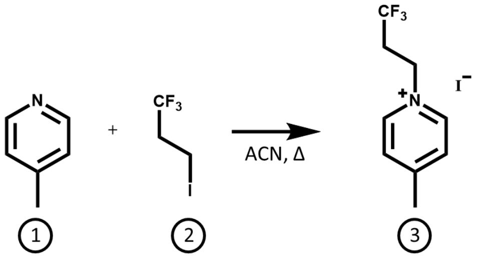

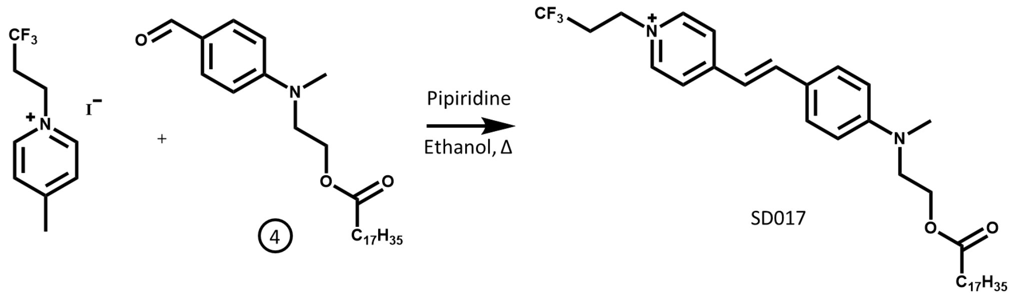

25 mmol of **1** was dissolved in 20 mL acetonitrile and placed into a round bottom flash with condenser fitted. To this solution 25 mmol of **2** in 20 mL acetonitrile was added dropwise. Following addition the solution was refluxed at 80 degrees °C under nitrogen for 24 hours. Solvent was removed via rotary evaporation and product (**3**) was recrystallized in ethyl acetate using a minimal amount of ethanol. ^{1}H NMR (400 MHz, CDCl3) δ 9.32 (d, J=6.64Hz, 2H), 7.88 (d, J=6.44Hz, 2H), 5.33 (t, J=6.23Hz, 2H), 3.11 (m, 2H), 2.73 (s, 3H). ^{19}F NMR (400 MHz, CDCl3) δ -63.93 (t, J=10.23Hz, 3F), Figure S13.

0.5 mmol of **3** and 0.426 mmol of **4** were dissolved in 20 mL of dry ethanol. 10 µL of pipiridine was added and the reaction was refluxed for 72 hours. Following this reaction solvent was removed via rotary evaporation and the dark red product (**SD017**) washed with cold ethanol. ^{1}H NMR (400 MHz, CDCl3) δ 8.96 (d, J=6.78Hz, 2H), 7.79 (d, J=6.88Hz, 2H), 7.64 (d, J=15.86Hz, 1H), 7.54 (d, J=8.97Hz, 2H), 6.88 (d, J=15.86Hz, 1H), 6.76 (d, J=9.02Hz, 2H), 5.09 (t, J=11.96Hz, 2H), 4.28 (t, J=6.01Hz, 2H), 3.70 (t, J=6.18Hz, 2H), 3.10 (s, 3H), 3.05 (m, 2H), 2.26 (t, J=7.53Hz, 2H), 1.57 (t, J=7.22Hz, 2H), 1.25-1.24 (m, 28H), 0.87 (t, J=6.86Hz, 2H). ^{19}F NMR (400 MHz, CDCl3) δ -63.99 (t, J=10.29Hz, 3F), Figure S14.

Additional Results

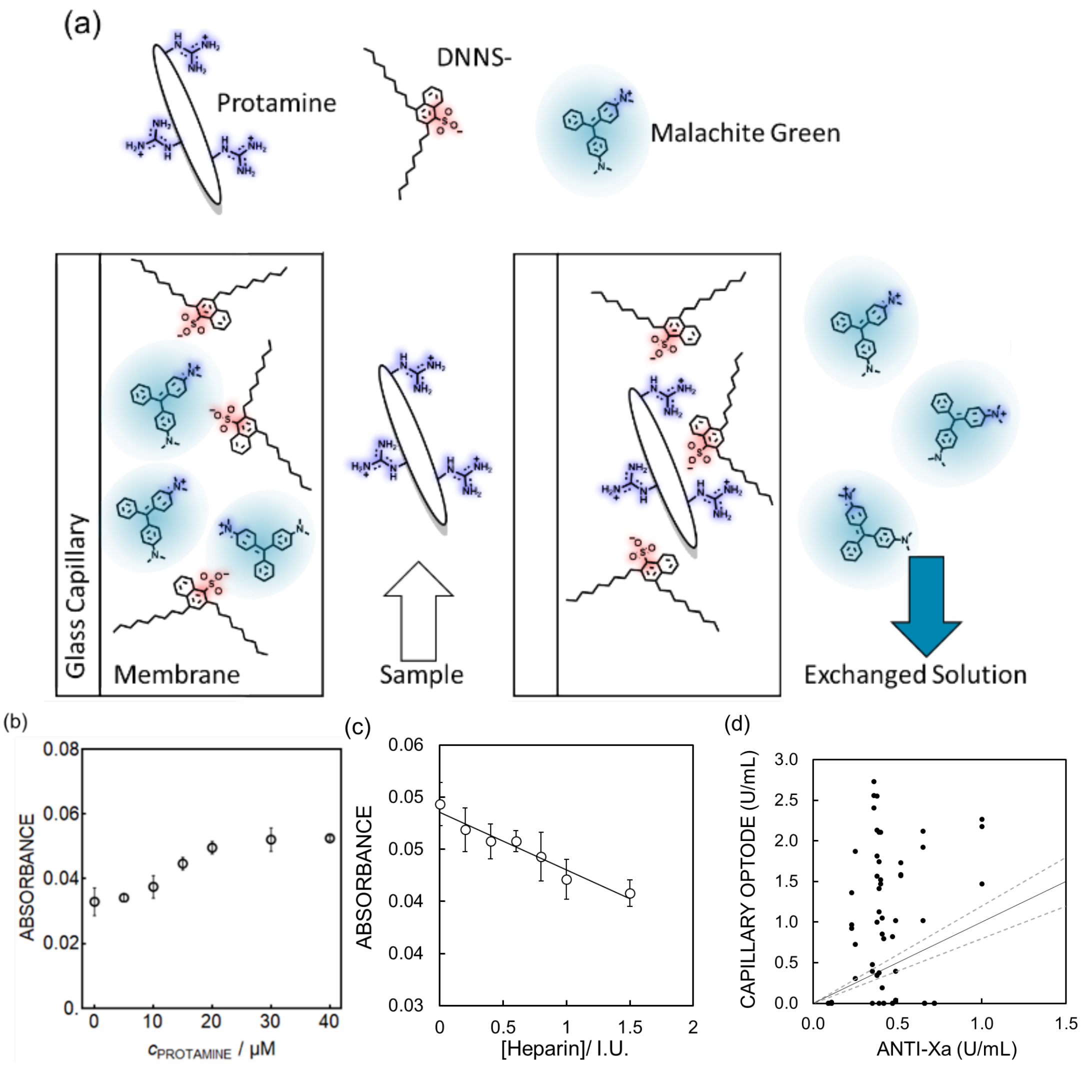

Figure S1. (a) Schematic illustration of the unsuccessful capillary-based protamine assay mechanism where protamine displaces the cationic Malachite Green dye upon extraction, increasing the absorbance of the solution. (b) Resulting protamine response of the capillary assay with 50 μL serum. (c) Capillary assay of varying concentration of heparin in serum by adding an excess of 15 μM protamine to the serum sample where protamine-heparin binding occurs. (d) Correlation plot for heparin concentration measured by the capillary and anti-Xa assay for 20 patient plasma samples. 15 μM protamine was added to each plasma sample and the heparin concentration was calculated based on the calibration curve shown in (c). Spearman correlation with one-tailed t-test gives parameters r=0.02 and p=0.45, indicating no correlation (n=3, ±SD).

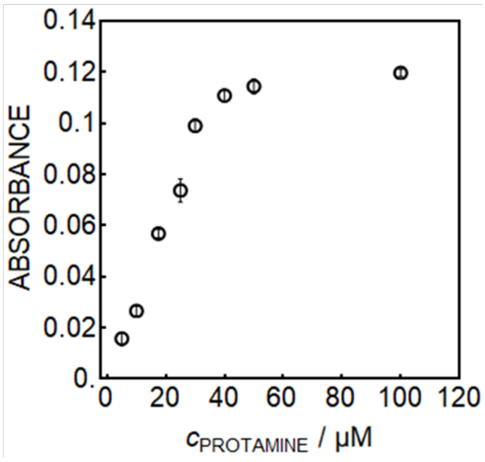

Figure S2. Response of the capillary-based sensor to protamine in the sample buffered by 10 mM universal buffer at pH7.4. 50 mL of the sample was aspirated into the capillary and brought into a contact with membrane optode. The sample solution was then incubated in the capillary for 5 mins and discharged into a cuvette containing 1 mL of deionized water for absorbance measurement (n=3 ±SD).

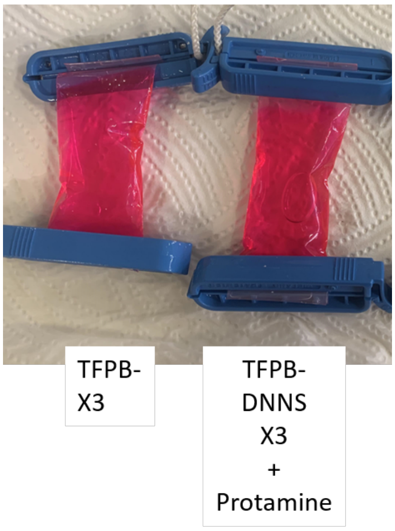

Figure S3. A picture of each nanoparticle solution after 20 h dialysis. Leftmost dialysis membrane contains nanoparticles having only $TFPB^-$ and $X3^+$ and the solution in the right one contains protamine sensors consisted of $DNNS^-$, $TFPB^-$ and $X3^+$ sensing components and 20 μM protamine. No color was observed in the dialysis solution.

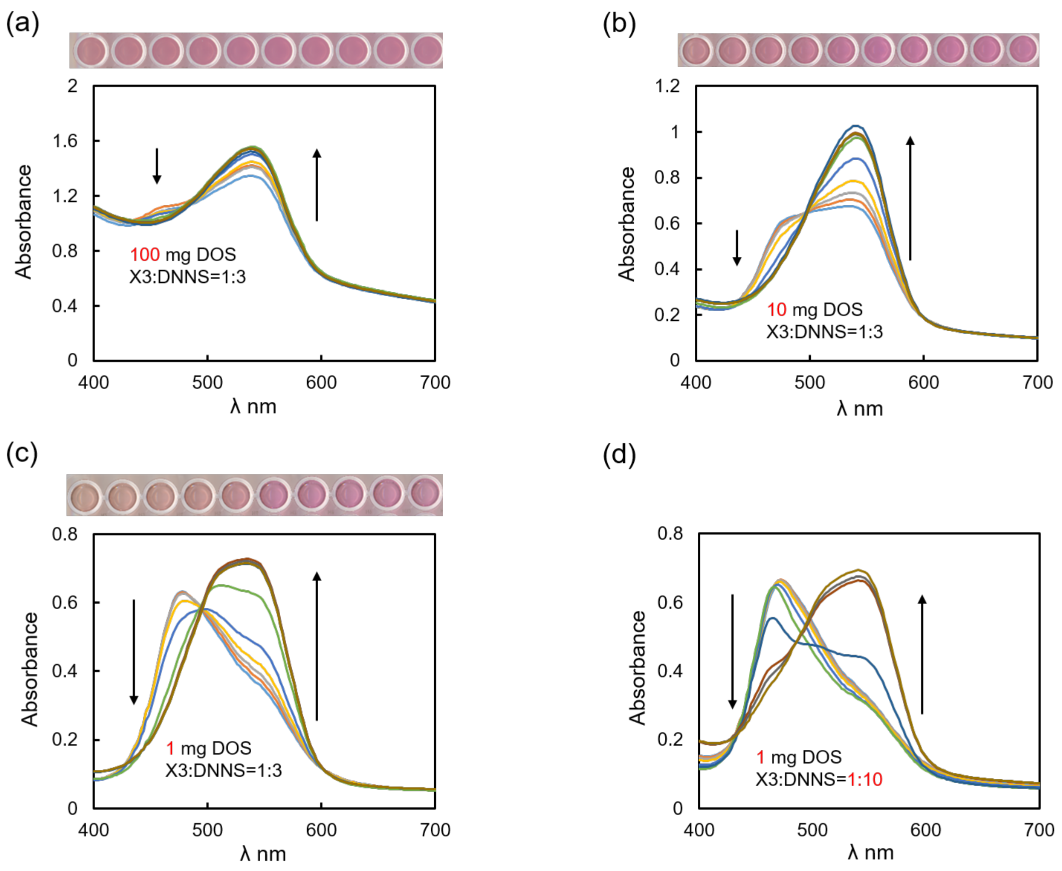

Figure S4. The absorbance response of nanosensors which works in the new sensing mechanism with different DOS (plasticizer) amount (a: 100 mg, b: 10 mg, c: 1 mg). (d) shows the response in the same condition as (c) except for the increased DNNS- amount.

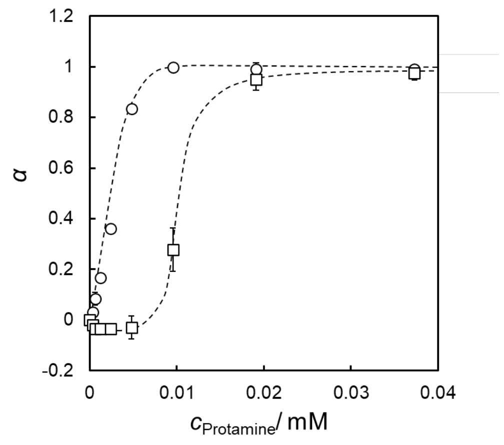

Figure S5. The protamine response of the sensors in Figure S4c (black circle, volume ratio; DOS : DNNS = 1 : 1.74) and the one in Figure S4d (black square, volume ratio; DOS : DNNS = 1 : 5.83) (n=3, ±SD).

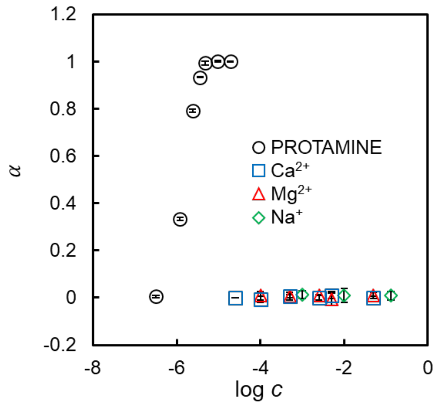

Figure S6. Response of the protamine nanosensor comprised of $DNNS^-$, $TFPB^-$ and $X3^+$ to protamine (black circle) and atomic ions (Ca^{2+} (blue square), Mg^{2+} (red triangle), and Na^+ (green diamond)) as interfering species (n=3, ±SD).

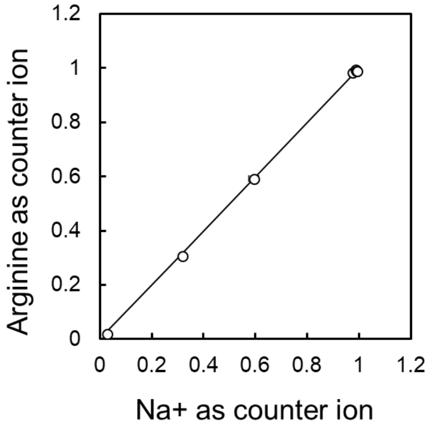

Figure S7. Reaction ratio (α) comparison of the developed nanosensors comprised of $DNNS^-$, $TFPB^-$ and $X3^+$ containing either Na^+ or arginine as a counter ion (n=3).

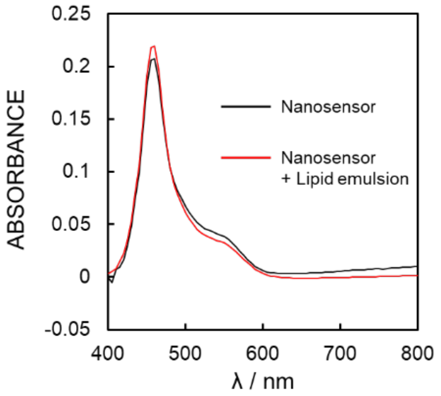

Figure S8. Absorbance signal of the protamine nanosensors in the presence (red line) and absence (black line) of cholesterol and triglycerides emulsified with phospholipid. The background absorbance of the lipid emulsion was subtracted the absorbance of the nanosensor/lipid emulsion mixture to obtain the red line.

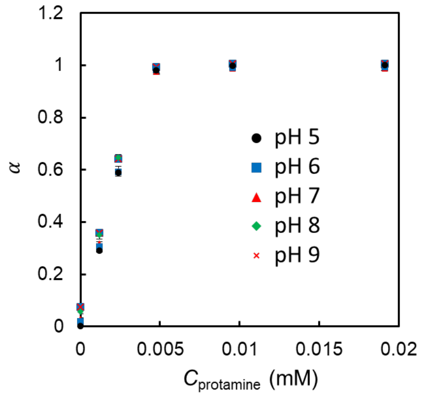

Figure S9. Response of the protamine nanosensors under different pH conditions. Universal buffer containing citric acid, boronic acid, monosodium phosphate and sodium hydroxide was used to adjust pH of the protamine standard solutions (n=3, ±SD).

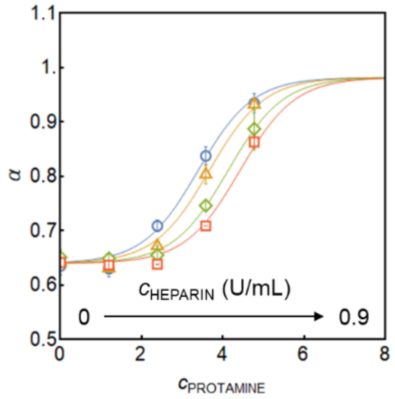

Figure S10. Protamine response of the nanosensors in pooled plasma containing 0 (blue circle), 0.3 (yellow triangle), 0.6 (green diamond) and 0.9 (red square) U/mL. U (unit) here is the international unit (n=3, ±SD).

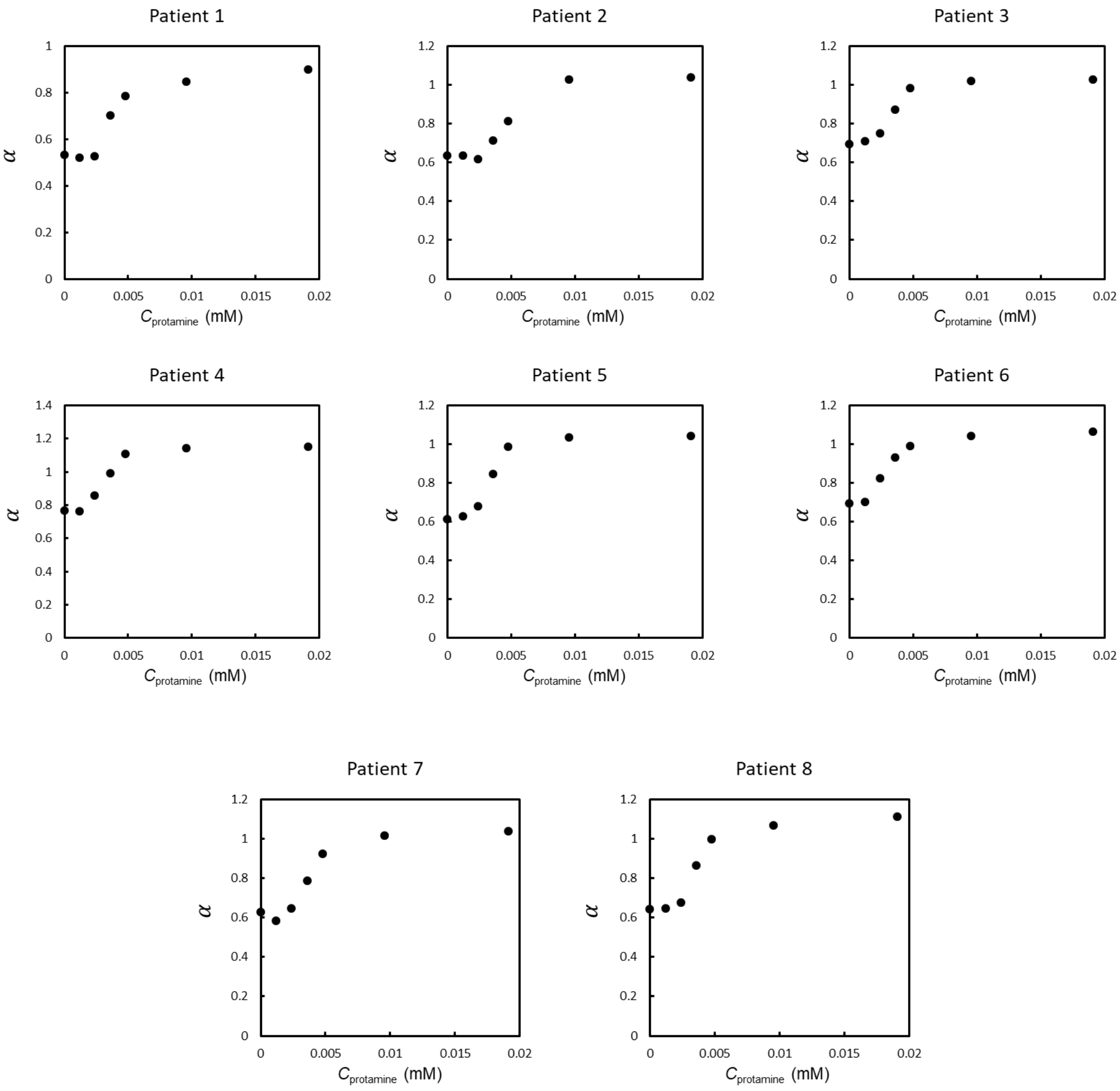

Figure S11. Protamine response curves obtained from patient samples (No. 1 - No. 8). The curve shifts in Figure S10 were used to calculate heparin levels for these patients.

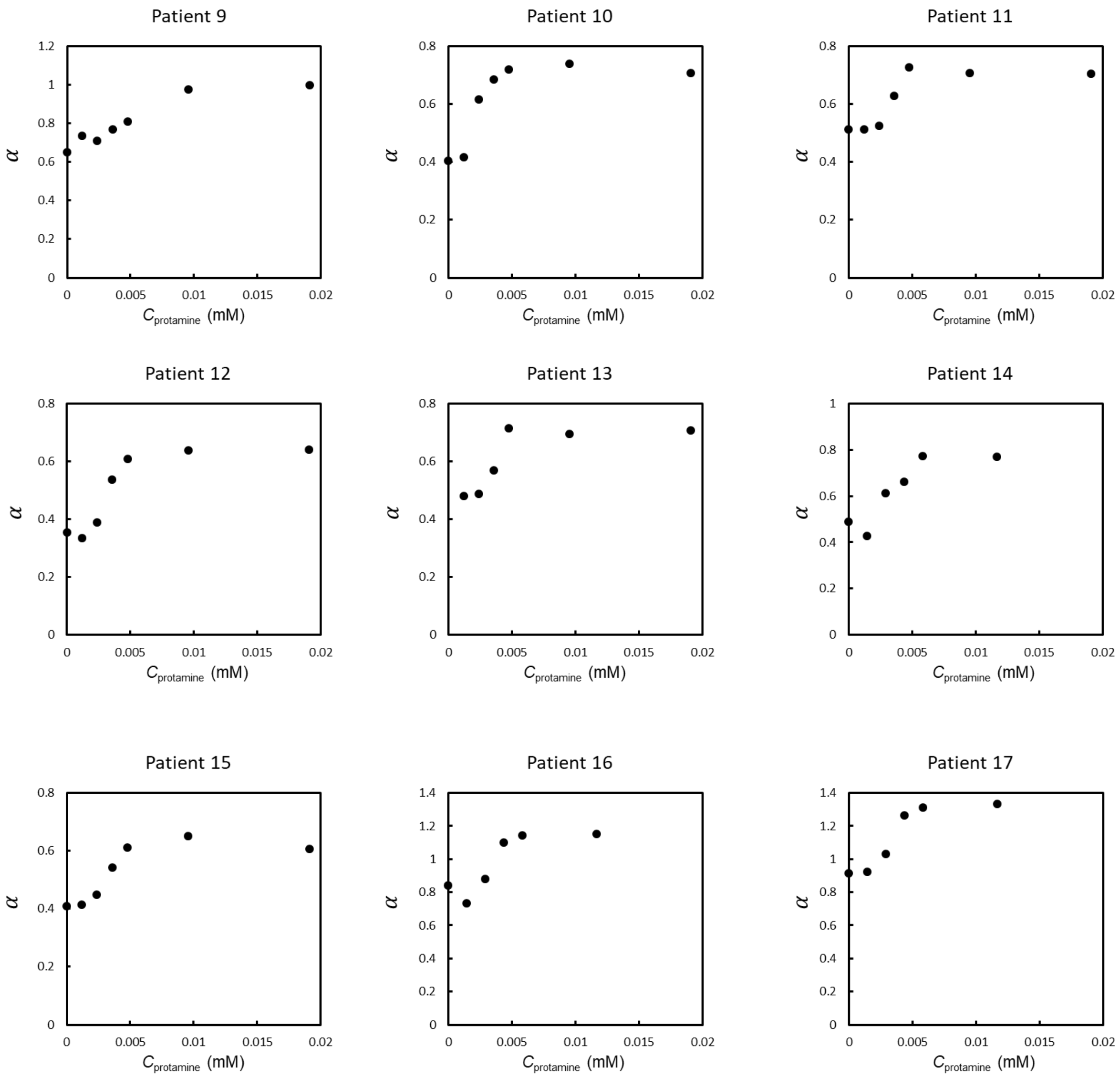

Figure S12. Protamine response curves obtained from patient samples (No. 9 - No. 17). The curve shifts in Figure 3a were used to calculate heparin levels for these patients.

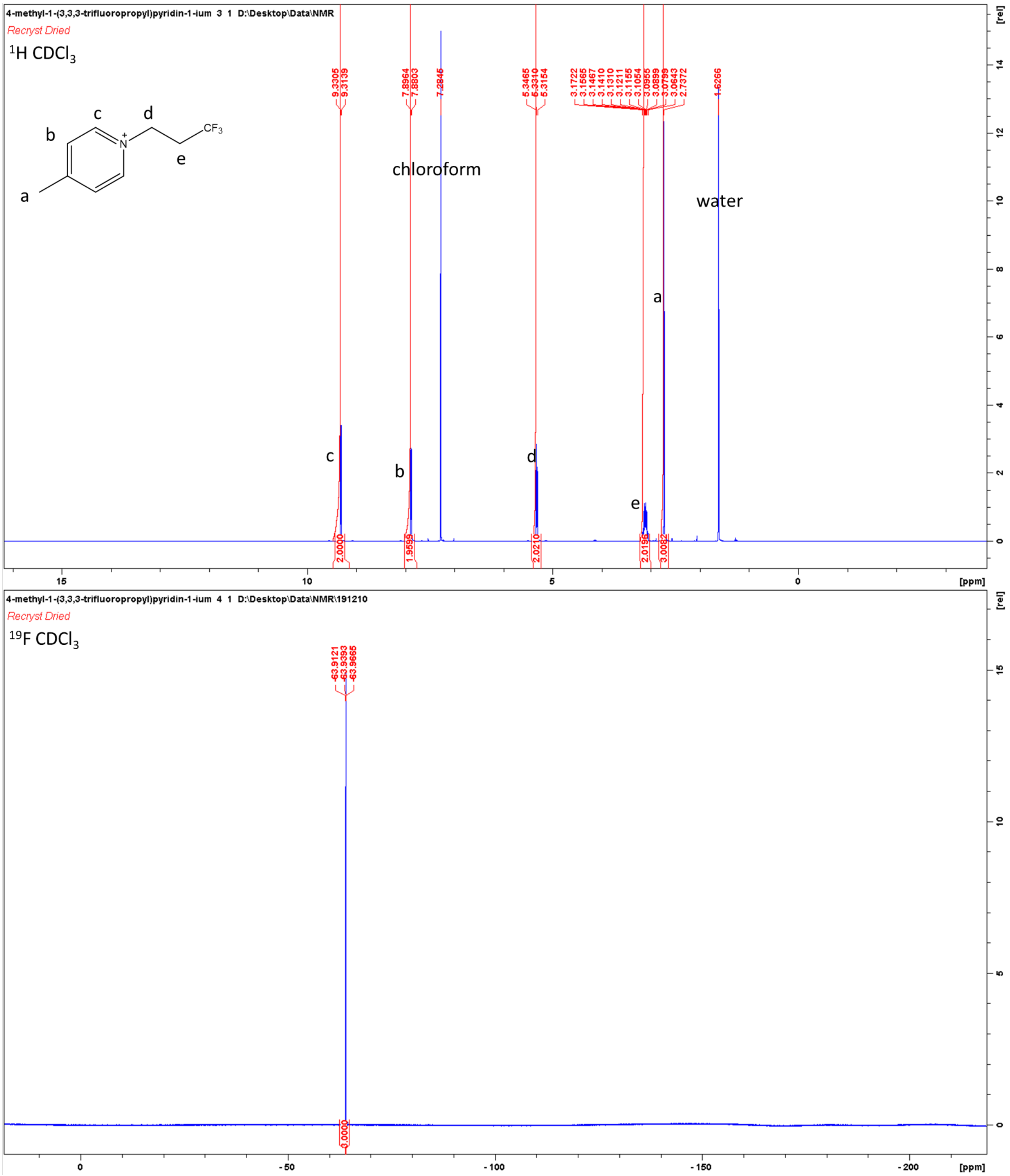

Figure S13: 1H and 19F 400MHz NMR of compound **3** (4-methyl-1-(3,3,3-trifluoropropyl)pyridin-1-ium).

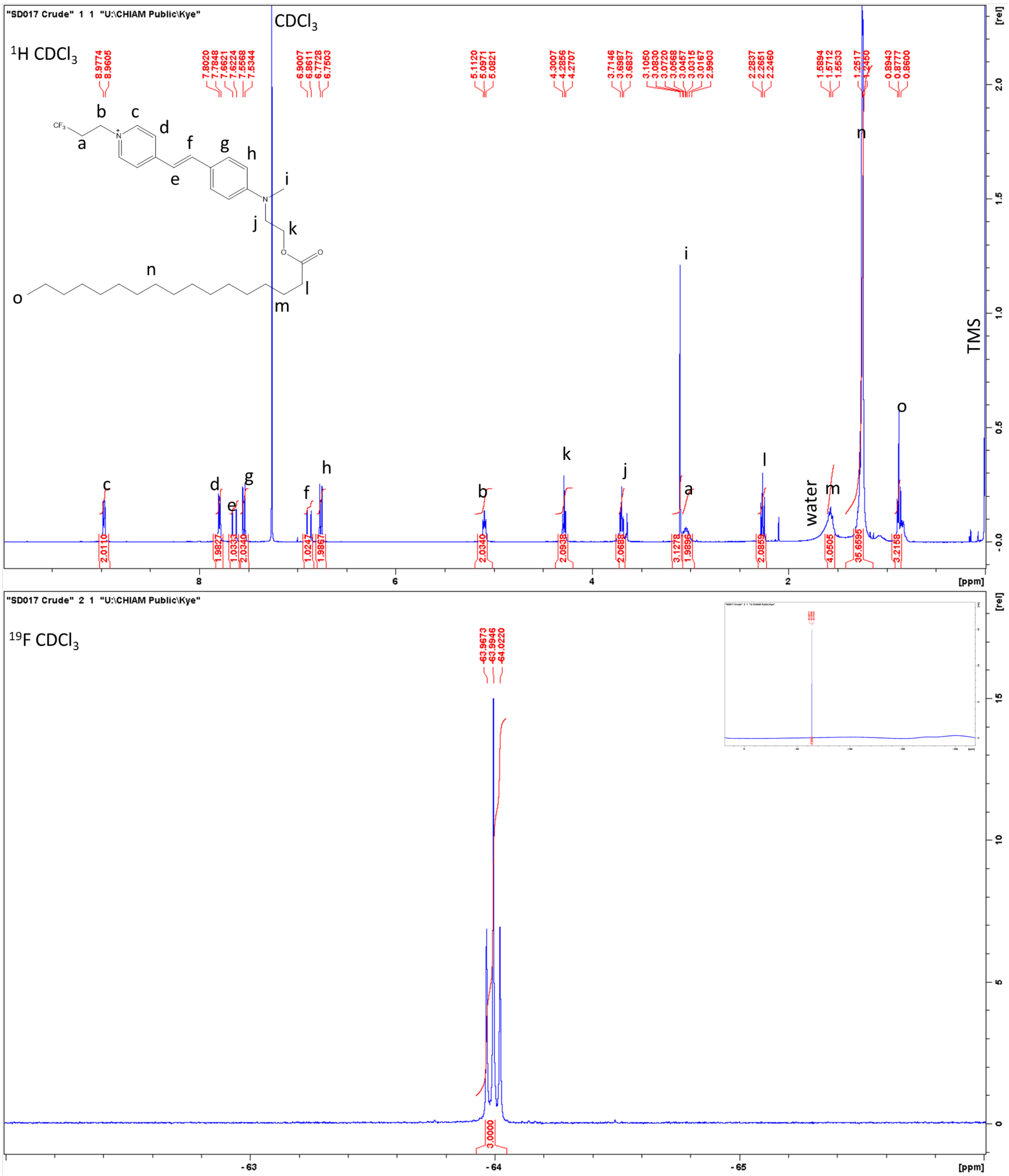

Figure S14: 1H and 19F 400MHz NMR of SD017.

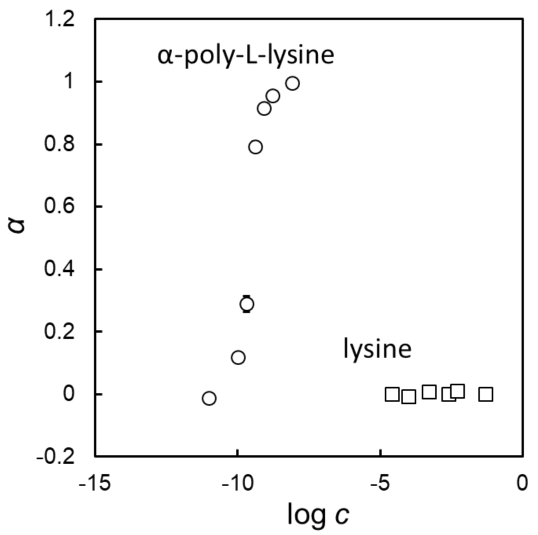

Figure S15: The response of the X3$^+$-based nanoparticle sensor to α-poly-L-lysine and lysine (n=3). Concentration of α-poly-L-lysine is regarded as 20,000 (n=3, ±SD).

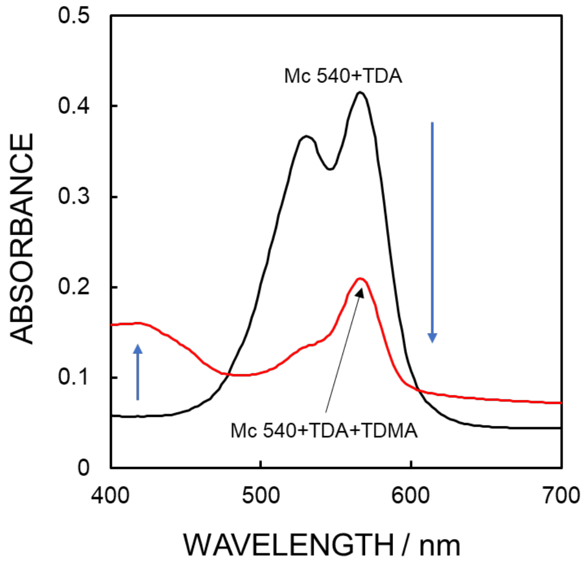

Figure S16: Figure 2. The absorption spectra of DOS-supported nanoparticles containing Merocyanine 540 (Mc540) and tetradocecylammonium (TDA) (black line) and the one containing Mc540, TDA and abundant tridodecylmethylammonium (TDMA) (red line).

Table S1. Zeta potential of the protamine sensor composed of $DNNS^-$, $TFPB^-$ and $X3^+$ in the absence and presence of protamine. Mean and deviation values were calculated from three repeated experiments.

Conditions	Zeta Potential
Nanosensor	-7.70±0.76
Nanosensor + 4 µM protamine	1.60±0.05
Nanosensor + 84 µM protamine	2.34±0.16

References

[1] X. Xie, A. Gutiérrez, V. Trofimov, I. Szilagyi, T. Soldati, E. Bakker, *Analytical Chemistry* **2015**, *87*, 9954-9959.
[2] L. Wang, X. Xie, J. Zhai, E. Bakker, *Chem. Commun.* **2016**, *52*, 14254-14257.
[3] Y. Soda, D. Citterio, E. Bakker, *ACS Sensors* **2019**, *4*, 670-677.

Author Contributions

Conceptualization, Y. S. and E. B.; Experiments, Y. S., R. N. and K. J. R.; Writing – original draft, Y. S. and K. J. R.; Writing – review and editing, Y. S., K. J. R. and E. B.; Supervision, E. B.

+Y. S. and K. J. R. contributed equally to this this work.
